# Life of Social Service

**Published:** 1920-04-24

**Authors:** 


### Life of Social Service.

Adeline, Duchess of Bedford, who died last week, lived a long life of social service, much of which was generously given in days before women's service was so common or so easy as at the present time. The main part of her social work was devoted to the cause of women. She supported with untiring zeal and consistency the Pimlico Association for Befriending Young Girls, but her chief work was for women convicts. She was the first of two lady visitors at Aylesbury Prison, and was a good friend and sound adviser to the unfortunate women prisoners. Her interest in all matters of prison administration was strong, and she devoted considerable attention to the ameliorative side of treatment for young offenders. Another point on which she could speak with authority was the care of the feeble-minded. She was also in close touch with the Queen Victoria Jubilee Institute for Nurses, and during the war she did valuable work as a member of the Joint Committee of the British Red Cross and St. John of Jerusalem. She was a member of the Government Committee appointed on behalf of British prisoners of war in Germany. Her war work was officially recognised by the bestowal of the Grand Cross of the Order of the British Empire. She was a devoted Churchwoman, and it is an interesting fact that, as one of the first ladies to take an active part in the Church Congress, she did much to break the convention against women on that platform. She was a gifted speaker.

